# Giant mucocele of the colon at the distal stump due to low-grade mucinous neoplasia

**DOI:** 10.1186/s40792-016-0223-9

**Published:** 2016-10-27

**Authors:** Toshiaki Tanaka, Kazushige Kawai, Hiroyuki Abe, Koji Murono, Kensuke Otani, Takeshi Nishikawa, Tomomichi Kiyomatsu, Keisuke Hata, Hiroaki Nozawa, Hironori Yamaguchi, Soichiro Ishihara, Masashi Fukayama, Toshiaki Watanabe

**Affiliations:** 1 Department of Surgical Oncology, University of Tokyo, 7-3-1 Hongo, Bunkyo-Ku, Tokyo, Japan; 2 Department of Pathology, University of Tokyo, 7-3-1 Hongo, Bunkyo-Ku, Tokyo, Japan

**Keywords:** Colonic mucocele, Low-grade mucinous neoplasia

## Abstract

We present the first ever report on a colonic mucocele observed at the distal stump of a transverse loop colostomy caused by neoplasia. A 37-year-old female consulted us because of abdominal pain and vomiting caused by cystic lesions in the upper left abdominal quadrant. A preoperative checkup revealed no sign of neoplastic lesions; however, tumor resection was performed because of the symptoms. The tumor was a mucocele of the distal stump of the transverse colon with obstruction interposed between the mucocele and stoma. Pathological diagnosis was low-grade adenoma; however, it appeared like low-grade mucinous neoplasia of the appendix rather than a normal colonic adenoma. The neoplasia existed in the transitional segment between obstruction and dilatation. As this is the first case of colonic mucocele caused by mucinous neoplasia, no definite consensus for diagnosis and treatment exists. With reference to low-grade mucinous neoplasia, we propose that complete surgical resection be performed for diagnosis and a favorable outcome.

## Background

Mucocele of the colon is a rare clinical manifestation, with only few reports being available. The etiology of colonic mucocele is yet to be unraveled, and no pathological features have been described so far. Here, we describe the first case of colonic mucocele caused by a low-grade colonic neoplasia, providing an insight on the clinical and pathological etiology of colonic mucocele.

## Case presentation

**Fig. 1:**
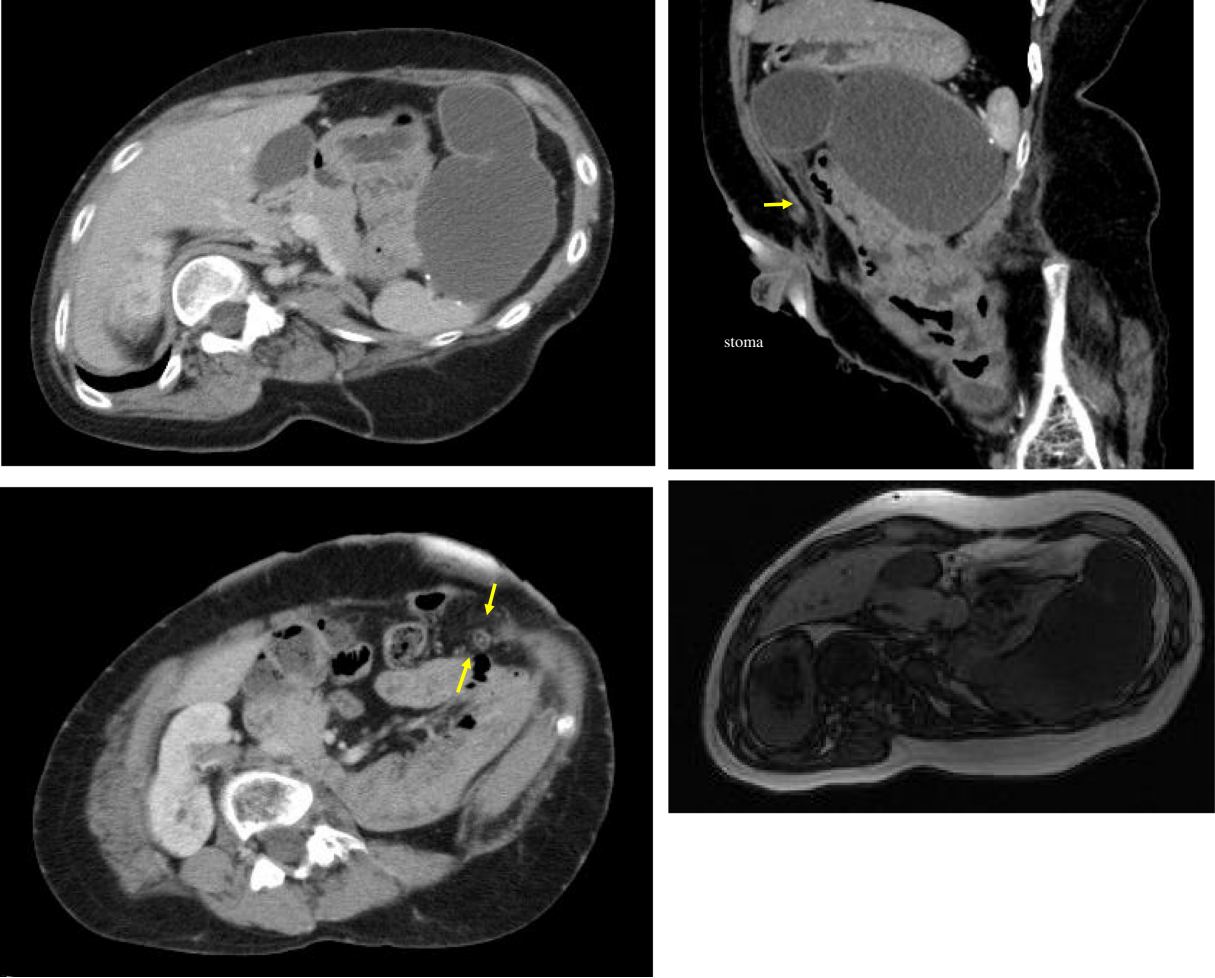
Contrast-enhanced CT image and MRI image of the tumor. Cystic and expansive tumor oriented between the pancreas, stomach, and spleen with no sign of invasion. The string-like structure interposed between the tumor and stoma. In the T1WI image, the cyst seemed to contain mucin and its different viscosities showed different strengths of MRI signals between the top and bottom of the cyst

A 37-year-old female with a history of frequent surgery consulted us because of recurrent vomiting and abdominal pain in the upper left quadrant. The first surgery, which was performed when the patient was 1 year old, was transverse loop colostomy for colonic obstruction due to a yolk sac tumor anterior of the sacrum; the tumor was subsequently resected when she was 2 years old. The following year, the tumor presented a local recurrence and was resected along with the sacrum below S4. Posterior pelvic exenteration for a complicated fistula between the vagina, rectum, and urinary bladder was performed when the patient was 8 years old. Radical cystectomy for hemorrhagic cystitis was performed the following year, and the patient was then subject to left nephrectomy for renal hypertension at the age of 12. Furthermore, several minor surgeries were performed; however, all operative records, including the ones referred to above, were not available. For the purpose of understanding the present anatomy and finding the cause of the symptoms, we performed diagnostic imaging. Contrast-enhanced computed tomography (CT) showed a large cystic tumor in the upper left abdominal quadrant, exhibiting expansive growth with no sign of invasion into the stomach, pancreas, or spleen—the organs surrounding the tumor. No sign of rupture was found. A thin string-like structure interposed between the tumor and the stoma was observed in the CT image (Fig. 1). In addition, no structures corresponding to the descending, sigmoid, or recto-sigmoid colon showed up in the CT images as they were probably removed in a past surgery. In MRI images (T1WI), the cystic lesion seemed to contain mucin, and its viscosity differed between the bottom and the top of the cyst, estimated by the different signal levels of MRI (Fig. 1). She underwent colonoscopy from stoma. The stenosis was located at 5 cm distal of the loop colostomy, and the lumen was so narrow that even the small-diameter endoscope (GF-240N Olympus Corporation, Tokyo, Japan) was impassable. We did not perform endoscopic biopsy because the endoscope did not reach the dilated lumen beyond stenosis. By percutaneous puncture, the tumor was seen to be filled with mucin, presenting a high concentration of carcinoembryonic antigen (CEA, 14,851 ng/mL); however, no malignant cells were found on cytological examination. We also observed elevated serum CEA levels (16.1 ng/mL). Contrast radiography from inside the cyst failed to show any connection to the stoma. We considered mucocele of the colonic stump, cystic lymphangioma of the retroperitoneum, or mucinous cystadenoma in a differential diagnosis. Surgical resection was performed because of clinical symptoms.

**Fig. 2:**
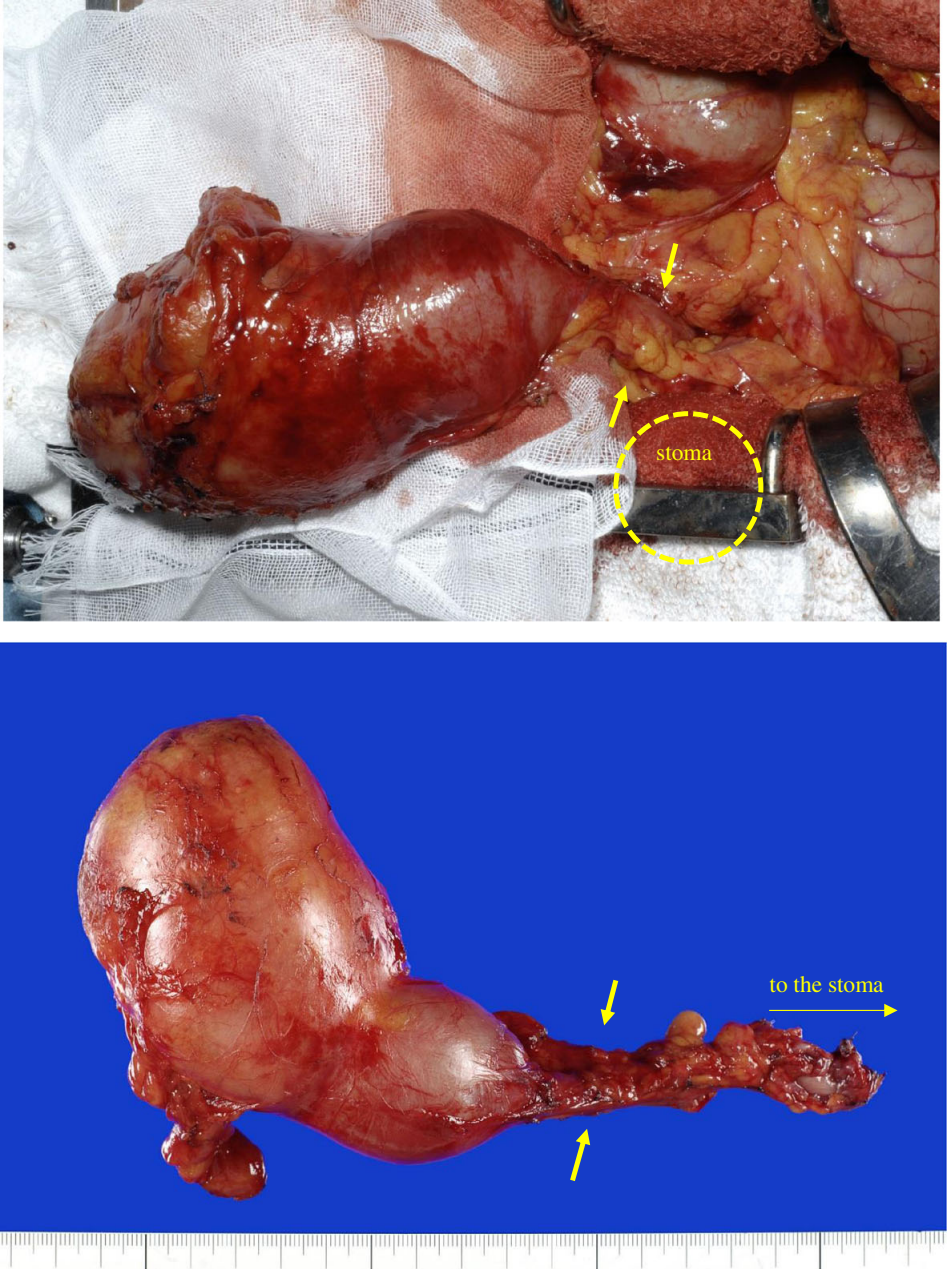
Cystic tumor as a colonic mucocele at the colonic stump distal to the colostomy. A strictured and atrophic colon (*arrows*) interposed between the dilated stump and colostomy that is invisible under the towel

Following upper midline abdominal incision, we found the transverse colon, which was distal to the site of loop colostomy, to be expanded indicating a cystic tumor. The segment between the loop colostomy and cyst appeared strictured; therefore, we speculated that mucin was trapped at the stump (Fig. 2). We detached the tumor from the surrounding organs, dissecting immediately below the stoma. The strictured colonic segment was completely removed.

**Fig. 3:**
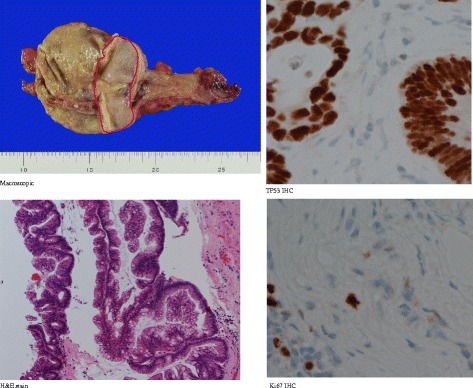
Neoplastic epithelium in the transitional region between the dilated colon and obstruction (marked with *red line*). It presented a different structure than common colonic adenoma, with pseudo-stratified nuclei and papillary-proliferating cells with mucin content. Immunohistochemical examination showed no excess expression of TP53. The Ki-67-positive positive rate was similar to the normal mucosa

Macroscopically, the cyst was filled with yellowish mucin, and no tumor change was found at the mucosal surface of the cyst and stricture segment. However, during microscopy (Fig. 3), dysplastic changes in the stricture were observed, the morphology of which appeared similar to that of low-grade appendiceal mucinous neoplasms because it showed pseudo-stratified nuclei, papillary-proliferating cells with mucin content, and loss of the lamina muscularis mucosae and the stroma in the lamina propria mucosae was replaced with fibrous tissue. No invasive carcinoma or severe atypia was found. Immunohistochemical examination showed neither overexpression of p53 nor abnormal distribution of Ki-67 stain.

The patient was discharged on the 12th postoperative day; the serum CEA level decreased to 3.3 ng/mL 1 month after the surgery.

### Discussion

Appendiceal mucocele, which is caused by the accumulation of mucin due to appendiceal obstruction, is a well-known type of appendiceal tumor, accounting for 0.1–0.3 % of all appendectomies [1]. They are often asymptomatic and incidentally found during surgery or diagnostic imaging for other diseases. Conversely, colonic mucocele is an extremely rare clinical manifestation, with only five cases reported so far, all of which were symptomatic because of large-sized lesions, and most of which developed following colonic surgery. Two cases developed upon diverting colostomy, with mucinous tumors developing distal to the stoma [2, 3]. The other two cases developed after ileosigmoid bypass as palliative surgery for malignant colonic obstruction, with the mucinous cyst developing at the isolated segment of the colon [4, 5]. In all cases, the unused bowel segment was the predilection site of mucin accumulation, similar to the present case. Leaving a bowel segment unused is a risk factor for the development of colonic mucocele.

The existence of neoplastic lesions similar to low-grade appendiceal mucinous neoplasia was a striking feature in our case. Low-grade mucinous neoplasia is often found in appendiceal mucocele; however, no such neoplasia has been reported so far in a patient with colonic mucocele. We hypothesized on how neoplasia could be related to the etiology of the mucocele of the present case. First, atrophic changes developed in the colon distal to the site of loop colostomy, resulting in narrowing of the luminal diameter. Even at this point, the intraluminal space would be preserved for continuous discharge produced by the distal stump. Subsequently, however, the neoplastic lesion produced more amounts of viscous mucin which then pass the stricture where the mucocele developed. This hypothesis presents a possible explanation for why the patient developed mucocele a long time after colostomy. Hence, the development of neoplasia was the primary cause of the mucocele.

We did not obtain definitive diagnostic results prior to the surgery. Although percutaneous aspiration biopsy is potentially accompanied with complications like hemorrhage or tumor dissemination, we did not obtain informative histological findings even after this procedure. No abnormal accumulation was found by positron emission tomography, and signs of neoplasia were not identified by colonoscopy. This diagnostic uncertainty was reminiscent of the challenges encountered when diagnosing appendiceal mucocele [6].

Because this is the first case of colonic mucocele accompanied by low-grade mucinous neoplasia, any consensus regarding appropriate operative procedures does not currently exist. According to the WHO classification, low-grade appendiceal mucinous neoplasia is a malignant tumor, and therefore, complete surgical resection is suggested. Hence, complete surgical resection appeared appropriate in the present case. Moreover, given the condition that it is difficult to obtain a definitive pathological diagnosis preoperatively, we should consider surgery for colonic mucocele even in the absence of proof of dysplasia. The postoperative course of appendiceal mucocele is typically characterized by recurrence presenting as intraperitoneal myxoma, and medical follow-up by CT scan every 6–12 months is recommended. With reference to this recommendation, we intend to perform a periodic follow-up of the colonic mucocele.

## Conclusions

In conclusion, we demonstrated colonic mucocele caused by low-grade mucinous neoplasia. Although standardized diagnostic criteria and surgical procedures do not exist as yet, complete resection for colonic mucocele to avoid rupture and unfavorable clinical outcomes should be performed.

## Consent

We obtained written informed consent for publication from the patient.
